# Analysis of ^G^γ-158(C→T) polymorphism in hemoglobin E/β-thalassemia major in Southern China

**DOI:** 10.1186/1756-8722-3-29

**Published:** 2010-09-07

**Authors:** Rong Rong Liu, Ming Yue Wang, Yong Rong Lai

**Affiliations:** 1 Department of Hematology, First Affiliated Hospital, Guangxi Medical University, China

## Abstract

**Background:**

The ^G^γ-158(C→T) polymorphism plays important function in the clinical variability of HbE/β-thalassemia. There is little known about ^G^γ-158(C→T) polymorphism in HbE/β-thalassemia major in Southern China. This study aimed to explore the association between HbE/β-thalassemia major and this polymorphism in Southern China.

**Methods and Results:**

The frequency of the ^G^γ-158(C→T) polymorphism has been evaluated in 32 patients with HbE/β-thalassemia major from Southern China. Further analysis of the ^G^γ-158(C→T) polymorphism revealed the prominent frequency of this polymorphic pattern among HbE/β-thalassemia major patients (65.63%). The presence of this polymorphism was strongly correlated with the increase of HbF synthesis.

**Conclusions:**

The frequency of the ^G^γ-158(C→T) polymorphism was relatively high in Southern Chinese patients with HbE/β-thalassemia major, often accompanying with high production of HbF. This feature appears to be different with reports in other races and regions.

## To the Editor

Hemoglobin E/β-thalassemia(HbE/β-thalassemia) is a common form of severe thalassemia syndromes in the Southern Chinese provinces[1]. Clinical manifestations of these patients range from nearly asymptomatic to severe β-thalassemia disease. The ^G^γ-158(C→T) polymorphism (**-**158 Xmn I ^G^γ-globin polymorphism) has been shown to be associated with the increased production of HbF and can strongly influence this heterogeneity of HbE/β-thalassemia[1-6]. The condition of the **-**158 Xmn I ^G^γ-globin polymorphism has been rarely reported in HbE/β-thalassemia majors from Southern China. The present study was to investigate the frequency of the **-**158 Xmn I ^G^γ-globin polymorphism and its association with high HbF level in HbE/β-thalassemia major patients of the Southern Chinese.

**Figure 1:**
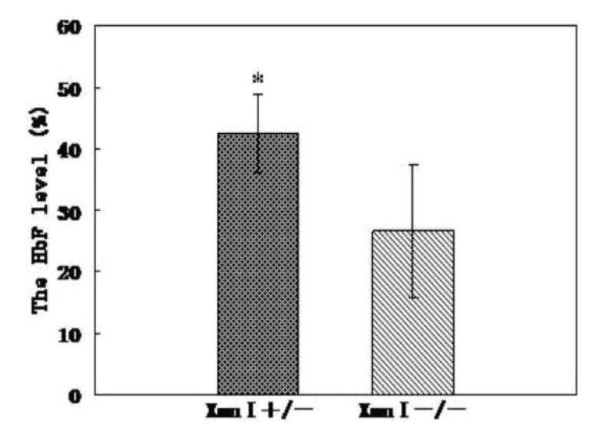
**The difference of HbF level in Xmn I +/- group and Xmn I -/- group among the HbE/β-thalassemia major.** The HbF level in Xmn I +/- group is obviously higher than in Xmn I -/- group (* P<0.01).

The clinical data were collected from 32 patients with HbE/β-thalassemia major who were seen at the First Affiliated Hospital, GuangXi Medical University. We also collected data from and compared with 30 unrelated healthy individuals. Table 1 shows the existence of the **-**158 Xmn I ^G^γ-globin polymorphism among HbE/β-thalassemia major and healthy controls. The frequency of polymorphism in HbE/β-thalassemia major (65.63%) was significantly higher than those in healthy controls (*P *< 0.00). In these patients, there were 6 β-thalassemia mutations detected in trans to the βE-thalassemia mutation. None of α-thalassmeia and homozygote of the -158 Xmn I ^G^γ-globin polymorphism were found in all samples. Fig 1. displays the association between the -158 Xmn I ^G^γ-globin polymorphism and HbF level among the HbE/β-thalassemia major. The HbF level in Xmn I +/- group was more than that in Xmn I -/- group, confirming the significant difference between these two groups. The analysis by Spearman correlation indicated that the **-**158 Xmn I ^G^γ-globin polymorphism was associated with increased HbF systhesis (r_p _= 0.588).

**Table 1:** Existence of the -158 Xmn I ^G^γ-globin polymorphism among 32 HbE/β-thalassemia major and 30 healthy controls

Polymorphism	Controls (%)	HbE/β-thalassmeia (%)
-158 Xmn I ^G^γ-globin	1 (3.33)	21 (65.63)
Xmn I +/+	0 (0)	0 (0)
Xmn I +/-	1 (3.33)	21 (65.63)
Xmn I -/-	29 (96.67)	11 (34.37)

In HbE/β-thalassemia, particularly in the major cases, during hematopoietic stress, point mutation at G-gamma promoter (the **-**158 Xmn I ^G^γ-globin polymorphism) can induce high gamma chain production rate[7]. The heavy hematopoietic stress from severe anemia may thus leads to the high frequency of this polymorphism in Southern Chinese patients with HbE/β-thalassemia major. This is the first report of the frequency of the **-**158 Xmn I ^G^γ-globin polymorphism in patients with HbE/β-thalassemia major in Southern China. These data suggest that screening of the **-**158 Xmn I ^G^γ-globin polymorphism and HbF level in early childhood may help on the management of HbE/β-thalassemia major patients and possibly prevent severe complications in Southern China.
